# Evaluation of a Phylogenetic Marker Based on Genomic Segment B of Infectious Bursal Disease Virus: Facilitating a Feasible Incorporation of this Segment to the Molecular Epidemiology Studies for this Viral Agent

**DOI:** 10.1371/journal.pone.0125853

**Published:** 2015-05-06

**Authors:** Abdulahi Alfonso-Morales, Liliam Rios, Orlando Martínez-Pérez, Roser Dolz, Rosa Valle, Carmen L. Perera, Kateri Bertran, Maria T. Frías, Llilianne Ganges, Heidy Díaz de Arce, Natàlia Majó, José I. Núñez, Lester J. Pérez

**Affiliations:** 1 Centro Nacional de Sanidad Agropecuaria (CENSA), La Habana, Cuba; 2 Universidad de las Ciencias Informáticas (UCI), La Habana, Cuba; 3 Centre de Recerca en Sanitat Animal (CReSA), UAB-IRTA, Campus de la Universitat Autònoma de Barcelona, 08193 Bellaterra, Barcelona, Spain; 4 Hospital Italiano de Buenos Aires, Juan D. Perón 4190, C1181ACH Buenos Aires, Argentina; 5 Departament de Sanitat i Anatomia Animals, Universitat Autònoma de Barcelona, 08193 Bellaterra, Barcelona, Spain; Kliniken der Stadt Köln gGmbH, GERMANY

## Abstract

**Background:**

Infectious bursal disease (IBD) is a highly contagious and acute viral disease, which has caused high mortality rates in birds and considerable economic losses in different parts of the world for more than two decades and it still represents a considerable threat to poultry. The current study was designed to rigorously measure the reliability of a phylogenetic marker included into segment B. This marker can facilitate molecular epidemiology studies, incorporating this segment of the viral genome, to better explain the links between emergence, spreading and maintenance of the very virulent IBD virus (vvIBDV) strains worldwide.

**Methodology/Principal Findings:**

Sequences of the segment B gene from IBDV strains isolated from diverse geographic locations were obtained from the GenBank Database; Cuban sequences were obtained in the current work. A phylogenetic marker named B-marker was assessed by different phylogenetic principles such as saturation of substitution, phylogenetic noise and high consistency. This last parameter is based on the ability of B-marker to reconstruct the same topology as the complete segment B of the viral genome. From the results obtained from B-marker, demographic history for both main lineages of IBDV regarding segment B was performed by Bayesian skyline plot analysis. Phylogenetic analysis for both segments of IBDV genome was also performed, revealing the presence of a natural reassortant strain with segment A from vvIBDV strains and segment B from non-vvIBDV strains within Cuban IBDV population.

**Conclusions/Significance:**

This study contributes to a better understanding of the emergence of vvIBDV strains, describing molecular epidemiology of IBDV using the state-of-the-art methodology concerning phylogenetic reconstruction. This study also revealed the presence of a novel natural reassorted strain as possible manifest of change in the genetic structure and stability of the vvIBDV strains. Therefore, it highlights the need to obtain information about both genome segments of IBDV for molecular epidemiology studies.

## Introduction

Infectious bursal disease (IBD), or Gumboro disease, is a viral infection that was described for the first time in the 60’s in Gumboro, Delaware, United States [1] and now occurs worldwide. The most important lesions observed in affected animals are lymphoid tissue damage found in the bursa of Fabricius [2].

IBD is caused by the IBD virus (IBDV), a non-enveloped virus belonging to the *Birnaviridae* family with a genome consisting of two segments of double-stranded RNA (segments A and B) [3]. Segment A (3.2 kbp) encodes a precursor polyprotein in a major open reading frame (ORF) that is cleaved by auto-proteolysis to yield the mature VP2 (outer capsid), VP4 (protease) and VP3 (inner capsid) proteins [4]. Segment B (2.8 kbp) encodes the virus RNA-dependent RNA polymerase (RdRp) VP1 [5] which exists in the virus particle both as a free protein and as a genome-linked protein; it interacts with the viral genome and the carboxy-terminal region of VP3 [6,7].

Two different serotypes of IBDV have been reported, which can be differentiated by virus neutralisation test [8]. All pathogenic isolates belong to serotype-1 strains [9,10]. Additionally, the serotype-1 strains, based on *in vivo* studies, molecular and phylogenetic analyses, have also been classified as: attenuated IBDV (atIBDV), classical virulent IBDV (cvIBDV), antigenic variant IBDV (avIBDV) and very virulent IBDV (vvIBDV) [11].

In particular, vvIBDV strains appeared in Belgium during the early 1980s associated with high mortality in young chickens [12,13]. Since vvIBDV strains emerged, they have been the source of great economic losses in the poultry industry in many countries. Whereby the scientific community has focused special attention related to the emergence and expansion of vvIBDV strains [14,15,16].

On the one hand, phylogenetic analyses have revealed independent evolutionary histories for the two genome segments (A and B), suggesting that a reassortment event may have played a role in the emergence of vvIBDV [14]. On the other hand, analyses based on reverse genetic have evidenced the fact that both genome segments influence in vvIBDV’s pathogenicity [17]. Hence, to conduct molecular epidemiology studies, including sequence analysis for both genomic segments, is an important step to explain the links between emergence, spreading and maintenance of the vvIBDV strains around the world.

Most of the phylogenetic studies based on segment A use the hyper-variable region of VP2 (HVR-VP2) as phylogenetic marker [18,19,20], whereas those few studies including segment B use the complete segment [21,22]. However, sequencing the full genome of IBDV is still expensive, from both computational and laboratory perspectives. Moreover, for many computationally intensive analyses, utilizing the full genome is unfeasible. It would be, therefore, beneficial to use only those genomic regions that contain the highest phylogenetic signal to reduce cost without losing valuable information [23,24].

In the current study, the reliability of a phylogenetic marker included into segment B (B-marker) was assessed. This B-marker could be applied in feasible molecular epidemiology studies involving both genome segments of IBDV. In addition, based on the reliability of the B-marker results and using phylogenetic inference based on HVR-VP2, the presence of a novel IBDV natural reassortant between segments A and B was reported.

The present work also highlights the need to obtain information about the genetic structure of both genome segments of IBDV, to elucidate the causes of the emergence and spreading not only of the vvIBDV strains but also of the novel IBDV natural reassortant (between segments A and B) strains. Novel IBDV natural reassortant strains have been described in few countries [25,26] and their effect on the epidemiological behavior of IBDV around the world remains unknown.

## Materials and Methods

### Samples

#### Ethics statement

International standards for animal welfare were used for all animal samples collected, following the regulations for animal sampling of the Institute of Veterinary Medicine (IMV), Ministry of Agriculture (MINAGRI) of the Republic of Cuba. The protocol was approved by the Committee on the Ethics of the MINAGRI of the Republic of Cuba and all efforts were made to minimize suffering of the animals. Birds were euthanized using cervical dislocation to collect the samples. The samples were sent directly from the IMV to the Animal Virology Laboratory at CENSA. The IMV is the official regulatory body of the Republic of Cuba; therefore, additional permits were not required.

#### Collection, selection and processing of samples

Forty-one bursa of Fabricius samples previously confirmed IBDV positive, which had been selected for molecular studies [16] were used in the current study. Additionally, the atIBDV strain used as vaccine was also included in the current work: the commercial vaccine strain “Gumboro” (Labiofam, S.A., Cuba) (http://www.labiofam.cu/productos/vacuna-gumboro.html) which is currently applied in the vaccination program conducted by Cuban veterinary services (Reviewed in [27]). The 41 bursal samples and the vaccine strain were printed on FTA cards, suited for the preservation of genetic material and adequate transportation [28]. The FTA cards were sent to *Centre de Recerca en Sanitat Animal*, *Barcelona*, *Spain* (CReSA) where the laboratory procedures were conducted.

### Laboratory procedure

The RNA of all 42 samples was extracted from FTA cards using the method described by Moscoso et al. [282] with modifications. Briefly: one gram from the spotted areas in the FTA cards was cut by a disposable scalpel blade and placed in 1.5 mL microcentrifuge tubes. For each FTA paper portion, 200 μL of nuclease free water were added, vortexed and incubated for 10 min at room temperature. The final suspensions were centrifuged at 7500g for 5 min at 4°C. RNA was extracted from 150 μL of supernatant recovered using Nucleospin RNA virus kit (Macherey-Nagel, Düren, Germany) following the manufacturer’s instructions. RNA was eluted in a final volume of 60 μL of nuclease free water.

The RNA extracted from FTA cards was used to amplify a region of the segment B using the primer pair GB2F:5´-GACCAGGAGTACTTCCCAAAR-3´/GB2R:5´- GTCCACTTGATGACTTGAGGT-3´, previously described by Lojkic et al. [29]. Within this region, the B-marker of 430 bp of length was selected, which is framed between N-terminal and F domains (S1 Fig). Both domains have a functional role in VP1 more than structural constrains [30]. The amplification products were visualized by electrophoresis on 1.8% agarose gels stained with ethidium bromide and were cleaned by QIAquick PCR purification kit (Qiagen Inc., Valencia, CA) following manufacturer’s instructions.

The resulting products were submitted to bi-directional DNA sequencing using a BigDye Terminator v3.1 cycle sequencing kit following the manufacturer’s directions (Applied Biosystems). Sequencing products were read on an ABI PRISM-3100 Genetic Analyzer (Applied Biosystems). The sense and antisense sequences obtained from each amplicon were assembled, and a consensus sequence for each gene was generated using the ChromasPro V1.5 program (Technelysium Pvt. Ltd., 2009). Nucleotide BLAST analysis (http://www.ncbi.nlm.nih.gov/blast/Blast.cgi) was initially used to verify the identity of each fragment sequence obtained. The sequences were submitted to the GenBank Database under accession numbers (LK022327–LK022343).

### Sequences dataset and multiple alignment

Different sequence datasets were organized: i) to assess the reliability of the B-marker, a set of 70 complete segment B sequences of IBDV available at GenBank Database (http://www.ncbi.nlm.nih.gov/) were used (Table 1, sequences denoted). From this sequences dataset two subsets were prepared: one containing the alignment of the entire B segments and the other one containing only the B-marker region. ii) To conduct the classification study, the Cuban sequences obtained were used together with reference strain sequences selected (Table 1, sequences denoted). For this study, sequences from the hypervariable region of segment A (HVR-VP2) were also used (S1 Table). Finally, iii) to estimate rates of nucleotide substitution per site, per year and the time to the Most Recent Common Ancestors (tMRCA) of specific groups, only sequences with a known year of collection were included. In all cases, the alignments of the sequences were performed using the algorithm ClustalW method included in the program BioEdit Sequence Alignment Editor [31].

**Table 1:** IBDV sequences used in the current study.

Accession number	Lineage	Strain	Year of collection	Country
**AB368971**	vvIBDV	N/A	N/A	N/A
**AF362770**	vvIBDV	N/A	N/A	N/A
**AF527038**	vvIBDV	N/A	N/A	N/A
**AF527040**	vvIBDV	N/A	N/A	N/A
**AJ318897**	vvIBDV	N/A	N/A	N/A
**AJ496637**	vvIBDV	N/A	N/A	N/A
**AM111354**	vvIBDV	N/A	N/A	N/A
**AY099457** ^*^	vvIBDV	T09	N/A	Nigeria
**DQ118374**	vvIBDV	N/A	N/A	N/A
**DQ166818**	vvIBDV	N/A	N/A	N/A
**DQ679812**	vvIBDV	N/A	N/A	N/A
**EU184686**	vvIBDV	N/A	N/A	N/A
**EU544150**	vvIBDV	N/A	N/A	N/A
**EU595674**	vvIBDV	N/A	N/A	N/A
**EU595675**	vvIBDV	N/A	N/A	N/A
**FJ695139**	vvIBDV	N/A	N/A	N/A
**GQ166971**	vvIBDV	N/A	N/A	N/A
**GQ449693**	vvIBDV	N/A	N/A	N/A
**JN982250**	vvIBDV	N/A	N/A	N/A
**KC109815**	vvIBDV	N/A	N/A	N/A
**NC_004179**	vvIBDV	N/A	N/A	N/A
**AF499930**	non-vvIBDV	N/A	N/A	N/A
**AY134875**	non-vvIBDV	N/A	N/A	N/A
**AY459321**	non-vvIBDV	N/A	N/A	N/A
**AY598355**	non-vvIBDV	N/A	N/A	N/A
**AY654284**	non-vvIBDV	N/A	N/A	N/A
**AY918947**	non-vvIBDV	N/A	N/A	N/A
**AY918949**	non-vvIBDV	N/A	N/A	N/A
**DQ403249**	non-vvIBDV	N/A	N/A	N/A
**DQ906922**	non-vvIBDV	N/A	N/A	N/A
**EF517529**	non-vvIBDV	N/A	N/A	N/A
**FJ040159**	non-vvIBDV	N/A	N/A	N/A
**GQ449689**	non-vvIBDV	N/A	N/A	N/A
**GQ449690**	non-vvIBDV	N/A	N/A	N/A
**GQ449691**	non-vvIBDV	N/A	N/A	N/A
**GQ449692**	non-vvIBDV	N/A	N/A	N/A
**GQ451331**	non-vvIBDV	N/A	N/A	N/A
**GQ452269**	non-vvIBDV	N/A	N/A	N/A
**JF811921**	non-vvIBDV	N/A	N/A	N/A
**JX134484**	non-vvIBDV	N/A	N/A	N/A
**JX134486**	non-vvIBDV	N/A	N/A	N/A
**JX682710**	non-vvIBDV	N/A	N/A	N/A
**JX682712**	non-vvIBDV	N/A	N/A	N/A
**KC603936**	non-vvIBDV	N/A	N/A	N/A
**M19336**	non-vvIBDV	N/A	N/A	N/A
**AF083093**	vvIBDV	IL3	N/A	Israel
**AF083094**	vvIBDV	IL4	N/A	Israel
**DQ679811**	vvIBDV	Hol	N/A	Netherlands
**AF362772**	non-vvIBDV	Cu-1M	N/A	Germany
**AF362748**	non-vvIBDV	Cu-1wt	1975	Germany
**X84035** ^*^	non-vvIBDV	P2	N/A	Germany
**AF092944**	vvIBDV	HK46	1994	Hong Kong
**AF240687** ^*^	vvIBDV	D6948	1987	Netherlands
**D49707** ^*^	vvIBDV	OKYM	1991	Japan
**X92761** ^*^	vvIBDV	UK661	1989	United Kingdom
**AF133905** ^*^	non-vvIBDV	Del-E	1985	United States
**AY029165** ^*^	non-vvIBDV	IM	1967	United States
**AY368654** ^*^	non-vvIBDV	GLS	1987	United States
**AY459320** ^*^	non-vvIBDV	Edgar	1967	United States
**U30819** ^*^	non-vvIBDV	OH	1982	United States
**EU162090** ^*^	non-vvIBDV	D78	1978	Netherlands
**AF322445** ^*^	vvIBDV	Tasik	N/A	Indonesia
**EU184688** ^*^	vvIBDV	Cro-Po/00	N/A	Croatia
**AF083092** ^*^	non-vvIBDV	2512	N/A	United States
**AF194429** ^*^	non-vvIBDV	CEF94	N/A	Netherlands
**AJ310186** ^*^	non-vvIBDV	CT	N/A	France
**EU184690** ^*^	non-vvIBDV	Cro-Pa/98	N/A	Croatia
**AB368969** ^*^	non-vvIBDV	KZC-104	2004	Zambia
**JQ411013** ^*^	non-vvIBDV	903/78	1978	Hungry
**EU162095**	non-vvIBDV	H30	2007	United States
LK022343^*^	to be determined	117/96PiR96	1996	Cuba
LK022327^*^	to be determined	BF3Hab97	1997	Cuba
LK022328^*^	to be determined	BF26Hab97	1997	Cuba
LK022329^*^	to be determined	61/98Hab98	1998	Cuba
LK022330^*^	to be determined	69/98Hab98	1998	Cuba
LK022331^*^	to be determined	135/00Hol00	2000	Cuba
LK022332^*^	to be determined	BF11Hab00	2000	Cuba
LK022333^*^	to be determined	45/02Hab02	2002	Cuba
LK022334^*^	to be determined	BF12Hab02	2002	Cuba
LK022335^*^	to be determined	BF29Hab04	2004	Cuba
LK022336^*^	to be determined	BF31Hab04	2004	Cuba
LK022337^*^	to be determined	BF14Cie08	2008	Cuba
LK022338^*^	to be determined	BF16Hab08	2008	Cuba
LK022339^*^	to be determined	BF19Hab09	2009	Cuba
LK022340^*^	to be determined	BF24Hab11	2011	Cuba
LK022341^*^	to be determined	BF25Hab11	2011	Cuba
LK022342^*^	to be determined	Gumboro_labiofam	2011	Cuba

Accession number in boldface: sequences selected to assess the reliability of the B-marker selected

* Sequences used for the classification study of the Cuban sequences

All sequences with an available year of collection were used in the time to the most recent common ancestors’ analysis.

N/A: not available.

### Model selection

The software jModelTest 2.0 [32] was used to estimate the best-fit model using the Akaike and Bayesian information criteria (AIC and BIC). The best-fit models for the complete segment B and phylogenetic marker were selected and used for phylogenetic analysis.

### Phylogenetic analysis

To remove sequences with a possible recombinant event from the alignment of all sequence datasets, searches for recombinant sequences and crossover regions were performed using Geneconv [33], RDP [34], MaxChi [35,36], Chimera [35], BootScan [37], SiScan [38], 3Seq [39] and LARD [40], all implemented in RDP3 Beta 4.1 [41]. Programs were executed with modified parameter settings determined according to the guidelines in the RDP3 manual for the analysis of divergent sequences (available upon request). Recombinant sequences were tested with a highest acceptable p value of 0.05, and Bonferroni’s multiple comparison correction was used. Analyses were conducted twice to ensure the repeatability of results.

Phylogenetic relationships of the IBDV strains were analyzed using the Bayesian Inference (BI) and Maximum Likelihood (ML) methodologies. Bayesian inference analyses were performed with the software MrBayes 3.1 [42,43]. The Markov chain Monte Carlo (MCMC) searches were run with four chains for 10 million generations, with sampling of the Markov chain every 100 generations. At the end of the run, the convergence of the chains was inspected through the average standard deviation of split frequencies and the first 25% of the trees were discarded. After discarding the burn-in, the four MCMC chains were combined and summarized on a majority rule consensus tree. The convergence was again assessed on the basis of the effective sampling size (ESS) using Tracer software version 1.4 (http://tree.bio.ed.ac.uk/software/tracer/). Only a log-likelihood with ESS’s of > 250 was accepted. A tree with clade credibility was constructed using the posterior probability distribution. The trees yielded from segment B and B-marker were unrooted. The tree yielded from segment A analysis was rooted using the sequence of IBDV serotype 2 with accession number at Genbank Database M66722.

ML trees were computed using the PHYML v3.0 [44], and confidence levels were estimated by 1000 bootstrap replicates.

### Comparison of topologies

All topologies were tested by the Kishino and Hasegawa test (K–H) [45] and the Shimodaira–Hasegawa test (S–H) [46], which computed the log-likelihoods per site for each tree and compared the total log-likelihoods for each proposed topology, using the PAMLv4.3 program [47]. Ten thousand replicates were performed using the K–H and S–H topologies tests by re-sampling the estimated log-likelihoods for each site (RELL model) [48]. Finally, the trees selected were visualized by FigTree v1.1.2 [49].

### Evaluation of the phylogenetic marker selected

#### Evaluation of the substitution saturation

The loss of phylogenetic information due to substitution saturation was evaluated comparing the complete segment B and the phylogenetic marker. The level of saturation was studied by plotting the pairwise number of observed transitions and transversions versus genetic distance. In addition, substitution saturation was evaluated with Xia’s test [23]. All these studies were performed using the DAMBE program [50].

#### Likelihood mapping

The phylogenetic signal of each sequence dataset was investigated by the likelihood mapping analysis of 100,000 random quartets generated using TreePuzzle [51]. In this strategy, if more than 30% of the dots fall in the center of the triangle, the data are considered unreliable for phylogenetic inference purposes.

#### Evaluation of the phylogenetic-relationship reconstruction

To assess if the B-marker selected contains the adequate phylogenetic signal to reduce cost without losing valuable information, topologies using complete segment B and the phylogenetic marker were constructed following the methodology described above. All topologies obtained using both datasets were compared as described above

### Substitution rates, time-scale of evolutionary history and phylodynamic analyses

The dataset of 31 sequences previously selected, was used to generate the BEAST input file by BEAUti within the BEAST package v1.8.1 [52] (freely available at http://beast.bio.ed.ac.uk). Rates of nucleotide substitution per site, per year and the tMRCA were estimated employing a Bayesian MCMC approach. The model selection was performed by estimating model marginal log-likelihood through the path sampling (PS) and stepping-stone (SS) sampling methods described by Baele et al. [53]. The estimation of model marginal log-likelihood through the PS and SS for the twelve coalescent demographic models including parametric models (constant population size, exponential growth and logistic growth) and nonparametric models (Bayesian skyline plot, BSP) with strict, uncorrelated lognormal distribution (UCLD) and uncorrelated exponential distribution (UCED) relaxed molecular clocks were calculated (S2 Table). Rates of nucleotide substitution per site, per year and the tMRCA were also estimated. In addition, a Bayesian skyline plot to infer the population dynamics in terms of changing levels of relative genetic diversity (Neτ) through time was performed. For BSP analysis data were collected and plotted using Graphpad Prism software 5.0 (1992–2007, Graphpad Prism software Inc.). An unpaired t test with Whelch’s correction was performed for statistical analysis of comparison of Neτ between groups.

In all cases the MCMC chains were run for 100 million generations, in order to obtain an ESS > 250, and the first 10% trees were discarded as ‘‘burn-in”, as recommended by the BEAST package Manual [54] (freely available at http://beast.bio.ed.ac.uk). Convergence was assessed by estimating the effective sampling size (ESS) after a 10% burn-in, using Tracer software version 1.5 (http://tree.bio.ed.ac.uk/software/tracer/). The tree with maximum log clade credibility was selected and visualized by FigTree v1.1.2 [48].

## Results

### Phylogenetic marker assessment

**Fig 1:**
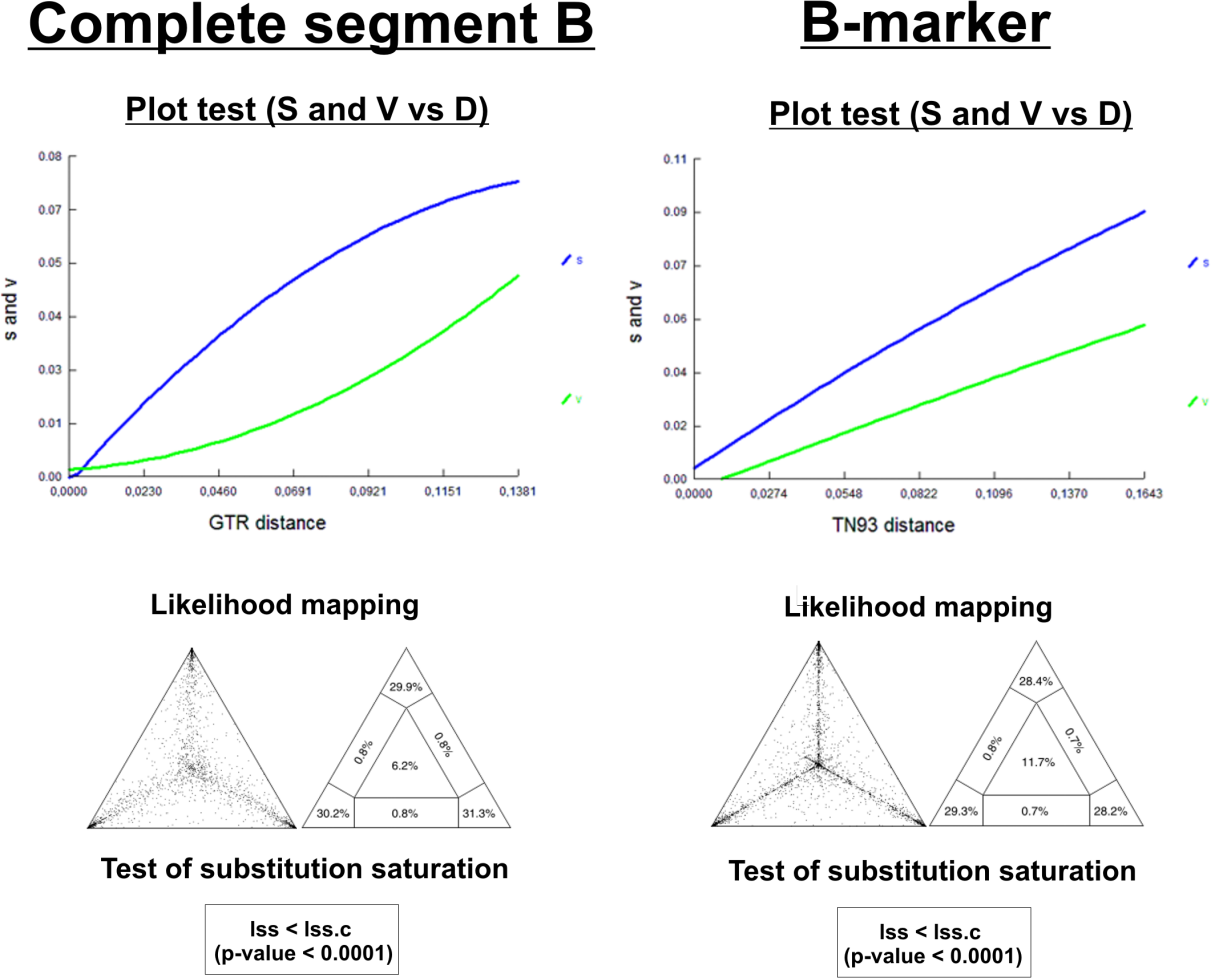
Comparison of the parameters: saturation of substitution and phylogenetic noise between complete segment B (left) and B-marker (right) for IBDV. On top, a plot representation of the number of transitions (S) and transversions (V) versus the genetic distance (D) calculated with the GTR model. Solid lines indicate the best fit to the observed data. In the middle, likelihood mapping of IBDV sequences, the dots inside the triangles represent the posterior probabilities of the possible unrooted topologies for each quartet. Numbers indicate the percentage of dots in the centre of the triangle corresponding to phylogenetic noise. At the bottom, the result of Xia’s test [49] and the statistical support for each sequence group is shown.

Saturation effects were investigated plotting the absolute number of transitions and transversions versus genetic distance for complete segment B and B-Marker (Fig 1). The number of observed transversions relative to that of transitions gradually increased with growing divergence, and both datasets resembled a line, indicating that transitions and transversions were not saturated. Moreover Xia’s test did not support saturation for complete segment B and the B-Marker (Iss < Iss.c, p < 0.0001) (Fig 1).

The phylogenetic noise in each data set was investigated by means of likelihood mapping. The percentage of dots falling in the central area of the triangles ranged from 6.2% for the complete segment B to 11.7% for the B-marker (Fig 1). None of the datasets showed more than 30% noise, which enabled the use of the B-marker to deduce the phylogenetic signal.

**Fig 2:**
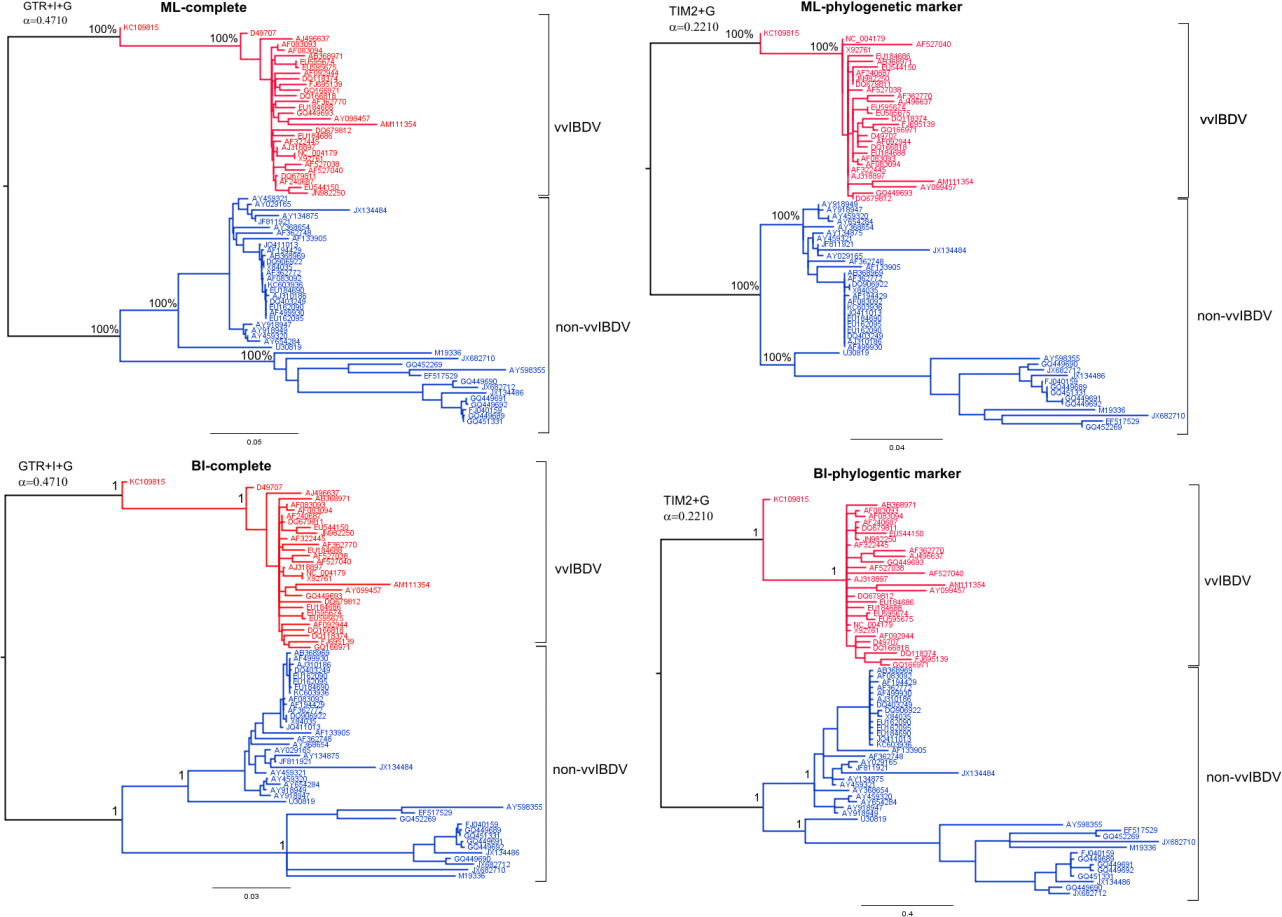
Topology comparison for IBDV. Topologies obtained from complete segment B (complete) and B-marker (phylogenetic marker) by Bayesian Inference (BI) and maximum likelihood (ML) analyses are shown. The model used to generate each tree is shown at the top left. Each main lineage is denoted (red for vvIBDV lineage and blue for non-vvIBDV lineage). Numbers along the branches refer to the bootstrap percentages and posterior probability values in the ML and BI analyses, respectively.

The phylogenetic relationships among the IBDV strains were reconstructed based on complete segment B and B-marker sequences by means of ML and BI analyses. Both algorithms yielded congruent results showing the same topologies, which was supported by moderate to high confidence values given by the bootstrap percentage and the posterior probability (Fig 2). Even though the ML tree yielded from complete segment B was the best, the statistical support for this tree was not significantly different from the BI tree from the complete segment B or the ML/BI trees from B-marker (S3 Table). Thus, all topologies obtained from both datasets yielded two highly divergent lineages, corresponding to both recognized vvIBDV and non-vvIBDV clades (Fig 2). Each lineage was strongly supported by the maximum posterior probability value of 1.00 and the highest bootstrap value of 100% (Fig 2).

### Phylogenetic analysis and molecular characterization

**Fig 3:**
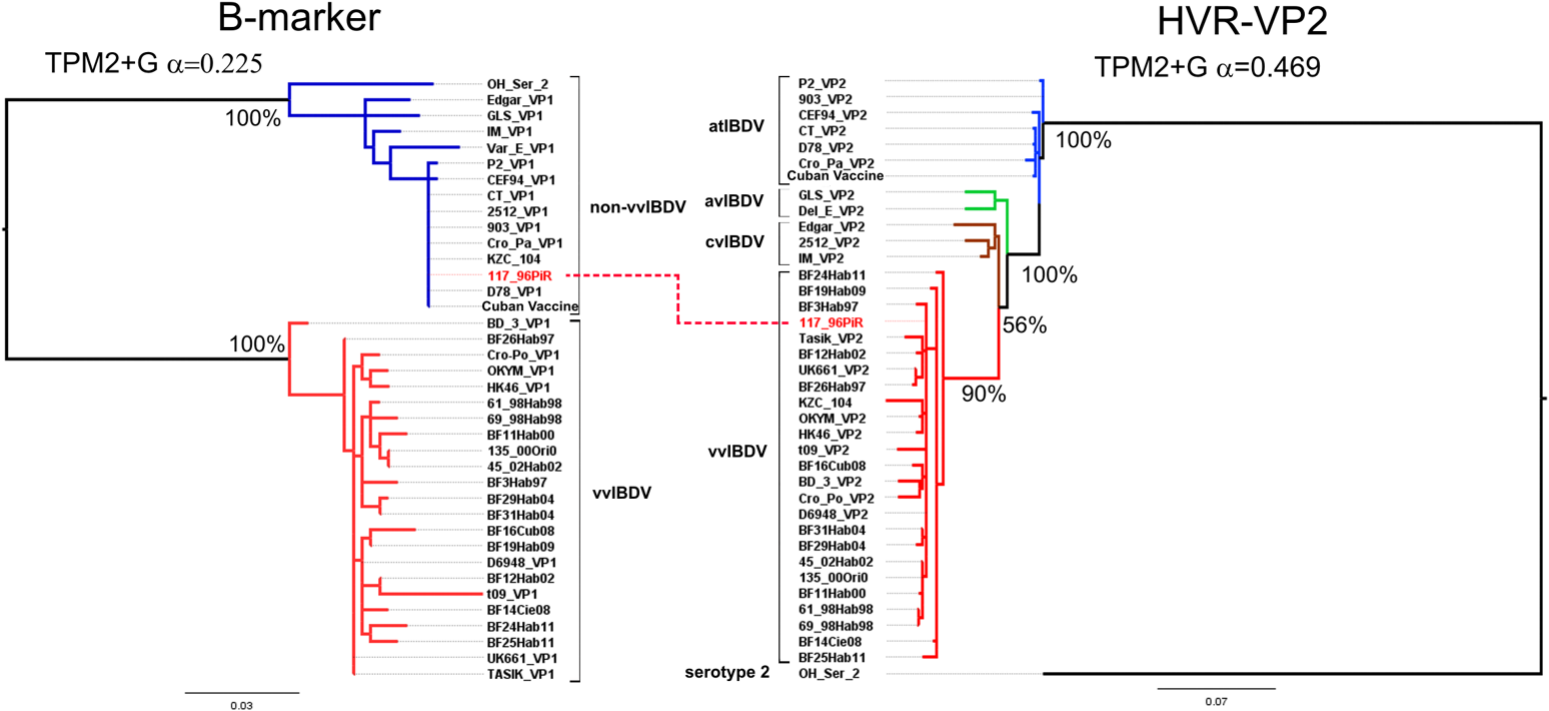
Phylogenetic trees of IBDV based on B-marker (left) and HVR-VP2 (right) sequences. The model used for phylogenetic inference is shown on top of each panel. Each lineage is denoted, for B-marker (vvIBDV lineage in red branches and non-vvIBDV lineage in blue branches), for HVR-VP2 (vvIBDV lineage in red branches, cvIBDV lineage in brown branches, avIBDV lineage in green branches and atIBDV lineage in light blue branches.) The Cuban IBDV natural reassortant between segment A from vvIBDV strains and segment B from non-vvIBDV strains was denoted with red letters.

The phylogenetic relationships among the IBDV strains were reconstructed based on B-marker sequences using ML analysis. Moderate to high confidence values given by the bootstrap percentage supported the topology yielded (Fig 3, left panel). The ML tree that estimated the phylogenetic relationships between the Cuban IBDV sequences and other reference IBDV strains is shown in Fig 3 (left panel). All Cuban IBDV field strains, excepting the Cuban vaccine strain and the 117/96Pir strain, were grouped in the defined lineage corresponding to the vvIBDV strains (Fig 3, left panel). Therefore, the Cuban vaccine strain and the 117/96Pir strain were grouped in the defined lineage corresponding to the non-vvIBDV strains (Fig 3, left panel).

At the same time, the topology yielded from HVR-VP2 showed that the Cuban vaccine strain was grouped within the defined cluster corresponding to atIBDV strains (Fig 3, right panel). Whereas, the remaining Cuban field strains were grouped within the defined cluster corresponding to vvIBDV strains (Fig 3, right panel). These Cuban field strains included the 117/96Pir IBDV, which had been grouped within the non-vvIBDV strain lineages for segment B (Fig 3). Thus, the Cuban 117/96Pir IBDV strain can be classified as natural segment-reassortant vvIBDV-like segment A and non-vvIBDV-like segment B.

Nucleotide and amino acid deduced comparisons were carried out among the sequences of the IBDV Cuban field strains obtained in the current study. The nucleotide sequence identities of the B-marker sequences among the 17 IBDV Cuban field strains ranged 87.2–100% and the deduced amino acid identities ranged 95.8–100% (Table 2).

**Table 2:** Nucleotides and deduced amino acid identities of Cuban IBDV strain based on B-marker region of segment B.

	nucleotides identity																	
	1	2	3	4	5	6	7	8	9	10	11	12	13	14	15	16	17	
1		0.986	0.986	0.981	0.983	0.979	0.983	0.981	0.983	0.986	0.979	0.972	0.983	0.974	0.976	0.881	0.881	1-BF3Hab97
2	0.986		0.99	0.986	0.988	0.988	0.988	0.99	0.988	0.99	0.988	0.981	0.993	0.983	0.986	0.883	0.883	2-BF26Hab97
3	0.986	1		0.99	0.993	0.988	0.993	0.986	0.988	0.99	0.983	0.976	0.988	0.979	0.981	0.876	0.876	3-61/98Hab98
4	0.986	1	1		0.988	0.983	0.988	0.981	0.983	0.986	0.979	0.972	0.983	0.974	0.981	0.876	0.876	4-69/98Hab98
5	0.979	0.993	0.993	0.993		0.99	1	0.983	0.986	0.988	0.981	0.974	0.986	0.976	0.979	0.874	0.874	5-135/00Hol00
6	0.979	0.993	0.993	0.993	1		0.99	0.979	0.981	0.983	0.976	0.969	0.981	0.972	0.974	0.879	0.879	6-BF11Hab00
7	0.979	0.993	0.993	0.993	1	1		0.983	0.986	0.988	0.981	0.974	0.986	0.976	0.979	0.874	0.874	7-45/02Hab02
8	0.986	1	1	1	0.993	0.993	0.993		0.983	0.986	0.988	0.986	0.993	0.983	0.986	0.886	0.886	8-BF12Hab02
9	0.979	0.993	0.993	0.993	0.986	0.986	0.986	0.993		0.997	0.981	0.974	0.986	0.976	0.979	0.876	0.876	9-BF29Hab04
10	0.986	1	1	1	0.993	0.993	0.993	1	0.993		0.983	0.976	0.988	0.979	0.981	0.879	0.879	10-BF31Hab04
11	0.986	1	1	1	0.993	0.993	0.993	1	0.993	1		0.981	0.99	0.981	0.983	0.888	0.888	11-BF14Cie08
12	0.986	1	1	1	0.993	0.993	0.993	1	0.993	1	1		0.988	0.974	0.976	0.881	0.881	12-BF16Hab08
13	0.986	1	1	1	0.993	0.993	0.993	1	0.993	1	1	1		0.986	0.988	0.881	0.881	13-BF19Hab09
14	0.972	0.986	0.986	0.986	0.979	0.979	0.979	0.986	0.979	0.986	0.986	0.986	0.986		0.983	0.883	0.883	14-BF24Hab11
15	0.972	0.986	0.986	0.986	0.979	0.979	0.979	0.986	0.979	0.986	0.986	0.986	0.986	0.986		0.872	0.872	15-BF25Hab11
16	0.972	0.972	0.972	0.972	0.965	0.965	0.965	0.972	0.965	0.972	0.972	0.972	0.972	0.972	0.958		1	16-Cuban vaccine
17	0.972	0.972	0.972	0.972	0.965	0.965	0.965	0.972	0.965	0.972	0.972	0.972	0.972	0.972	0.958	1		17-117/96PiR96
	amino acid identity																	

Triplet amino acids of positions 145/146/147 and the amino acid of position 242 in VP1 have been linked to change in IBDV virulence [55,56]. Therefore, comparisons of amino acid replacements based on this pattern were performed among all Cuban sequences included in the present work (S2 Fig). Thus, the pattern of the triplet amino acids **TDN** (145–147) present in vvIBDV strains was found in the Cuban field strains BF26Hab97, 61/98Hab98, 69/98Hab98, 135/00Hol100, BF11Hab00, 45/02Hab02, BF29Hab04, BF31Hab04, BF14Cie08, BF16Hab08, BF19Hab09, BF24Hab11 and BF25Hab11 (S2 Fig). The pattern of the triplet amino acids **NEG** (145–147) present in non-vvIBDV strains was found in the Cuban vaccine strain and the field strain 117/96Pir (S2 Fig). The field strain BF3Hab97 showed a unique pattern **SDS** (145–147) for this triplet amino acids (S2 Fig). Regarding amino acid of position 242, the Cuban field strains BF3Hab97, BF26Hab97, 61/98Hab98, 69/98Hab98, 135/00Hol100, BF11Hab00, 45/02Hab02, BF29Hab04, BF31Hab04, BF14Cie08, BF16Hab08, BF19Hab09 and BF25Hab11 showed the signature **242E** present in vvIBDV strains (S2 Fig), while the Cuban vaccine strain and the field strains 117/96Pir and BF24Hab11 showed the signature **242D** present in non-vvIBDV strains (S2 Fig).

### Evolutionary rates and tMRCA

PS and SS analyses based on B-marker showed an exponential coalescent and an exponential, uncorrelated clock best fitted to our data (S2 Table). The estimated mean (95% HPD) for the substitution rate of the segment B of all populations assessed of IBDV was 7.09x10^-4^(2.56x10^-4^-1.37x10^-3^) substitutions/site/year (Table 3). Nevertheless, both lineages showed different substitution rates for segment B, the estimated mean (95% HPD) of the substitution rate for non-vvIBDV lineage was 1.88x10^-4^(1.11x10^-6^-5.18x10^-4^) substitutions/site/year, and the mean of the substitution rate for vvIBDV lineage was 4.80x10^-3^(1.34x10^-4^-8.99x10^-4^) substitutions/site/year (Table 3). Thus, the substitution rate of segment B for vvIBDV lineage was approximately 40 times higher than the substitution rate of segment B for non-vvIBDV lineage.

**Table 3:** Estimated substitution rates and time to the most recent common ancestor (tMRCA) for the IBDV strains segment B.

Dataset of IBDV	Rate (s/s/y) (HPD95%)	tMRCA (y) (HPD95%)
Whole population	7.09x10^-4^(2.56x10^-4^-1.37x10^-3^)	1879 (1710–1965)
vvIBDV	4.80x10^-3^(8.99x10^-4^-1.34x10^-3^)	1981 (1965–1987)
Non-vvIBDV	1.88x10^-4^(1.11x10^-6^-5.18x10^-4^)	1917 (1810–1965)

(s/s/y): substitution per site per year

**Fig 4:**
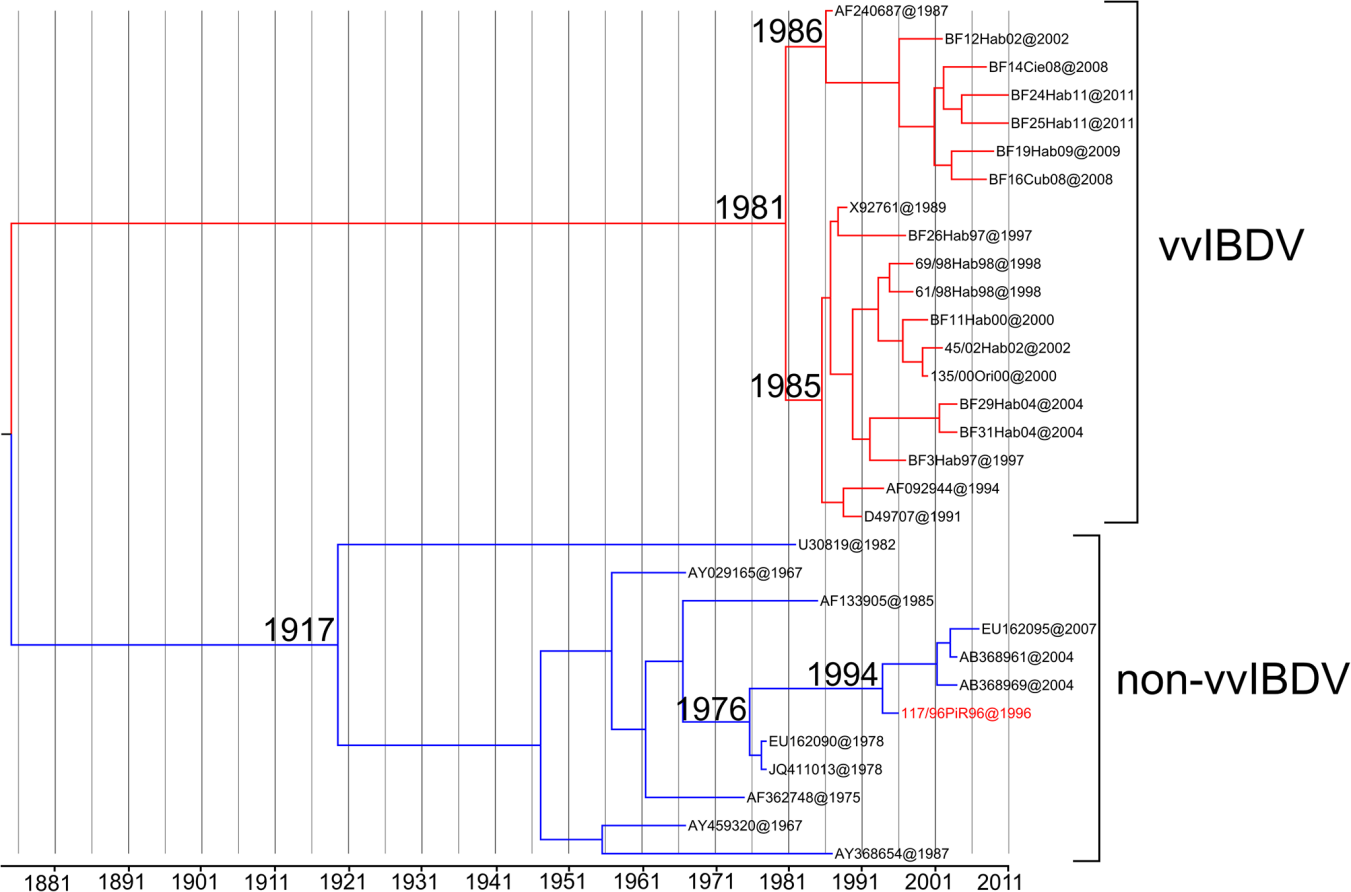
Maximum clade credibility (MCC) tree describing the evolutionary history for segment B of IBDV. The branches belonging to the main lineages were highlighted, in red for vvIBDV and blue for non-vvIBDV. The most probable year for the MRCA within each lineage was also denoted. The Cuban IBDV natural reassortant strain was also denoted with red letters, the most probable year for the MRCA for this strain was also denoted.

The date Bayesian phylogenetic tree obtained for the global IBDV strains was characterised by a clear temporal structure; the oldest samples tended to fall closer to the root of the tree, while the most recent samples were located at the most distal tips. The mean tMRCA for diversification of both lineages for segment B of IBDV was framed in different dates. The diversification of the non-vvIBDV lineage was located at approximately 1917 (95% HPD from 1810 to 1965), while the mean of tMRCA for vvIBDV lineage was 1981 (95% HPD from 1965–1987) (Fig 4). In this context, the ancestor for the cluster in which Cuban strain 117/96Pir96 was located was framed around the year 1976, whereas the ancestors for the remaining Cuban strains were defined between the years 1985 and 1986 (Fig 4).

### Phylodynamic

**Fig 5:**
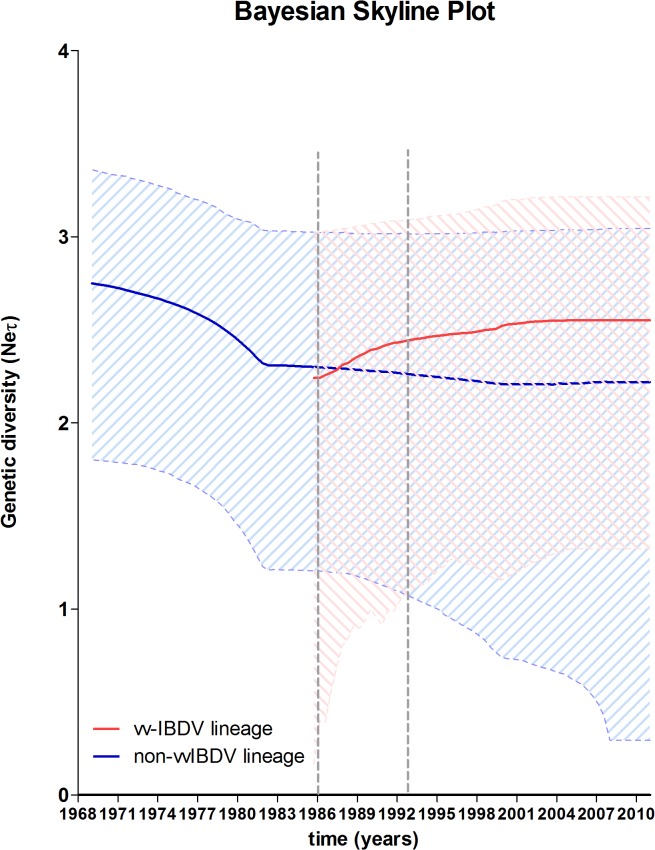
Demographic history of the global circulation of segment B for vvIBDV and non-vvIBDV lineages using Bayesian skyline plot. The y axes of the skyline plots represent relative genetic diversity (Neτ) expressed as Log (Neτ). The x axis represents the number of years prior to the date of the most recent isolate in each lineage. The thick colored line is the mean estimate (red for vvIBDV lineage and blue for non-vvIBDV lineage), and the 95% HPD are indicated by the shaded area. Grey dashed lines frame the abrupt increase in Neτ for vvIBDV lineage, coinciding with the expansion of vvIBDV strains to Western Europe (Belgium) in 1987, Africa (Egypt) around 1990, East Asia (China and Japan) around 1993 and the Caribbean Region (Cuba) around 1991–1993 (see [16] for details).

Demographic inference using the BSP model is summarised in Fig 5, which essentially plots Neτ as a function of time. Neτ can be considered a measure of relative genetic diversity that reflects the number of effective infections established by the virus. The BSP for both IBDV lineages showed different patterns for Neτ, indicating different epidemiological behaviours for both viral populations (Fig 5). For non-vvIBDV lineage, a decrease in Neτ from its emergence to early ‘80s was observed, with a subsequent maintenance in the Neτ, suggesting stability in the diversity of this population. On the contrary, an abrupt increase in Neτ from the emergence of vvIBDV lineage (approximately in 1986) to 1993 was observed (Fig 5), which proves an epidemic behaviour of this viral population during this period. Subsequently, a mild increase in Neτ was observed until year 2001 followed by a maintenance until 2011, suggesting a stability in diversity of this population. Despite of the stability of Neτ for vvIBDV lineage, the genetic diversity was statistically higher for this lineage than for the non-vvIBDV lineage (Fig 5).

## Discussion

The sudden and dramatic emergence of vvIBDV strains, which have caused high mortality rates in birds and considerable economic losses in different parts of the world since more than two decades [57], still represents a considerable threat to the poultry industry. The polygenic nature of IBDV pathogenicity, in particular the clear role of both segments (A and B) in vvIBDV’s pathogenesis [17,58], has shown that the characterization of IBDV strains based on both genome segments is essential to understand the epidemiological behavior of this viral agent. Nonetheless, the complete sequencing of both segments is impractical in routine practice in diagnostic laboratories. In addition, utilizing the full genome for molecular epidemiology studies would result in costly computational analyses, which would be also unfeasible [59]. Therefore, the use of phylogenetic markers for molecular epidemiology studies of IBDV is a useful and needful approach.

In the present study, a novel phylogenetic marker (B-marker) included into segment B of IBDV’s genome is proposed. The initial selection of B-marker was based on two main aspects: length of the fragment and location. Being the B-marker less than 500bp (430bp) of length, this fragment can be easily obtained and sequenced in laboratories with limited resources. Likewise, the size of the B-marker facilitates the computational analyses, avoiding delayed results generated by large size fragments (Reviewed in: [24]). In addition, B-marker is framed between N-terminal domain and partial F domain of VP1 [30]. The N-terminal domain of IBDV VP1 folds into a mixed α/β structure. This domain has been associated with the protein priming process and interacts with the fingers and thumb domains [60]. The F domain has been linked to the nucleotide recognition and binding process as well as to the RNA template binding mechanism [30]. Other domains such as domain D, starting from residue 252 (end of B-marker) are involved in structural integrity, and C-terminal domain is highly conserved not only among IBDV strains but also for all members of *Birnaviridae* family [30]. Thus, the structural domains in which B-marker is framed have a functional role in VP1 more than structural constrains; therefore, this region could be more influenced by evolutionary changes than possible negative selective restrictions.

The reliability of this B-marker to conduct feasible molecular epidemiology studies involving both genome segments of IBDV was also evaluated. Furthermore, the global phylodynamic of the IBDV strains was studied based on the consistency of the results obtained for this B-marker. In this context, the genetic diversity of the Cuban IBDV strains was also analyzed. Besides, the current work revealed the presence of a novel IBDV natural reassortant between segment A from vvIBDV strains and segment B from non-vvIBDV strains in Cuban IBDV population.

Reliable reconstruction of phylogenies using molecular data can be affected by several factors; one of these critical factors is the saturation of substitutions [61]. When a dataset is saturated, phylogenetic reconstruction may be misled by homoplasious signal [23], which is evidenced by no further increase in transitions observed despite increasing genetic distance, indicating that multiple substitutions at nucleotide positions have occurred. The gradual increase of transitions/transversions observed with respect to genetic distance for B-marker suggests a lack of saturation for this selected region. Correspondingly, Xia’s test supported the same outcome. Xia’s test is based on the ratio of observed entropy to the entropy of full substitution saturation defined as the index of substitution saturation (*I*_*SS*_). If this value is not significantly smaller than the critical I_SS_ (*I*_*SSC*_) (value at which the sequences start to fail to recover the correct tree), the sequences have experienced severe substitution saturation and should not be used for phylogenetic reconstruction [23]. From the results obtained for B-marker we can infer that this region does not experience substitution saturation and can be used for phylogenetic studies.

An important aspect for a successful phylogenetic experimental design is to predict the power of a dataset. Phylogenetic noise from fast-evolving sites misleads phylogenetic inference [62]. Therefore, identification of optimal levels of noise exclusion reduces the number of topologies that are not significantly worse than the optimal tree and allows a more robust inference of phylogeny and stronger conclusions about character evolution [63]. In our study, the phylogenetic noise associated to the B-marker proposed was just around 4% less, in comparison with whole segment B. This result strongly supports the use of the region proposed as phylogenetic marker since a value less than 30.0% for phylogenetic noise is accepted as reliable in phylogenetic inference [64,65].

A high consistency between some particular regions and their whole genome in referring phylogenetic relationships can be considered as a good signature of a phylogenetic marker [66]. As a whole, we consider that the B-marker is a reliable phylogenetic marker for IBDV strains since it was able to reconstruct the same tree as the complete segment B of the viral genome. This outcome was also supported by S-H test, which has been proved as powerful indicator of the optimal level of phylogenetic noise reduction and topologies exclusion [62].

Thus, the B-marker was able to discriminate between the defined lineages corresponding to the vvIBDV and non-vvIBDV strains. Taking advantage of the reliability of this phylogenetic marker, the Cuban IBDV strains were classified for this segment of the viral genome. Previously classified vvIBDV strains [16] were also classified as vvIBDV for segment B.

Using the B-marker we also estimated the emergence of vvIBDV-lineage in the global scenario around 1981. This date matches the range of years proposed by Hon et al. [14], who determined the emergence of tMRCA for the vvVP1 sequences in the same year. In our previous work, the expansion of strains carrying HVR-VP2 sequences linked to high virulence of IBDV was fixed starting from Iran in 1981 [16]. These results clearly suggest that around 1981, two important events took place. Firstly, the emergence of the genetic background very virulent for both segments (A and B) of IBDV. Subsequently, these two genetic backgrounds very virulent were put together into a reassorted strain that we know today as vvIBDV.

The demographic history of the segment B of the IBDV genome for non-vvIBDV lineage, showed a trend toward a decrease in genetic diversity, possibly generated by the introduction of effective vaccination programs against classical and low virulent strains from early stages of the discovery of the disease [67,68]. Taking into account that most commercially available conventional live IBDV vaccines are based on classical virulent strains (Reviewed in [69]), the use of vaccine strains with similar genetic background to the field strains could have subjected IBDV’s population to repeated bottleneck effects leading to a loss of fitness through a process of natural selection [70,71].

On the contrary, the demographic history of the segment B of IBDV genome for vvIBDV lineage showed a trend toward an initial growth of genetic diversity, possibly generated by the initial emergence of these strains. Conventional live IBDV vaccines based on classical virulent strains exhibit only poor efficacy against vvIBDVs [69]. Thus, IBDV strains with this new genetic background had the possibility to express the potential advantages of their large *quasispecies* cloud making expedite their establishment and spreading around the world, especially during the first years of their emergence. The maintenance in genetic diversity of segment B for this lineage, suggesting stability in diversity of this population during 2001–2011, could be associated to the application of more strict control measures for these emergent strains during this period. In fact, novel vaccines were developed to be more effective against vvIBDV strains, such as subunit vaccines, genetically engineered live IBDV vaccines, DNA vaccines and others [69]. However, different factors including reversion to virulence of the vaccine strains [69], non-sufficient induction of protective immune response [72] as well the intrinsic property of IBDV to evolve quickly, have made the task of controlling IBD by vaccination even more challenging.

The expansion of vvIBDV strains was linked to a reassortment event of the genome (segment B) of IBDV with a mutant VP2 background, which caused a sudden increase in virulence of these kind of strains [14]. Thenceforth, vvIBDV strains have been kept antigenically and genetically homogeneous, spreading to most of the countries (with the exception of Australia) at least for two decades. However, the recent isolation of vvIBDV strains with rare natural segment-B-reassorted [25,26] have evidenced a possible change in the genetic structure and stability of vvIBDV strains. Thereby, Ingrao et al. [73] suggested that it is probably only a matter of time until vvIBDVs are replaced by an emerging strain with new antigenic or pathotypic properties. In fact, He et al. [25] have currently reported that reassortant IBDV strains were dominantly prevalent in southern China during 2000–2012 [25].

In the current work, the strain 117/96Pir was defined as a natural reassortant between segments A from vvIBDV and B from non-vvIBDV. The date of the emergence of this natural reassortant in Cuba was estimated to be 1994, after the introduction of vvIBDV strains to Cuba [16]. In fact, the ancestor for the cluster in which Cuban strain 117/96Pir96 was located was framed around the year 1976, coincident with the first introduction of the virus in the country [16]. Therefore, the reassortment event that originated the strain 117/96Pir96 seems to have occurred between the novel vvIBDV strains introduced in Cuba around 1991 and the atIBDV strains, which had been circulating among the Cuban poultry population since 1977.

The pathogenicity and antigenicity of the strain 117/96Pir reassortant is unknown. Even though, several studies have confirmed that IBDV reassortants and vvIBDV strains possess different biological characteristics [58,74]. The pathogenicity and antigenicity of the strain 117/96Pir reassortant need to be further investigated. Additional pathogenicity and antigenicity studies would also be required for BF3Hab97 and BF24Hab11 strains. On the one hand, BF3Hab97 strain showed a unique pattern of triplet amino acids 145–147, which has been associated with pathogenicity of IBDV [54]. On the other hand, BF24Hab11 strain showed the signature **242D** present in non-vvIBDV strains suggesting a possible attenuation [29]. Therefore, both strains could be useful to better understand the signatures and virulence factors for IBDV.

Regarding the evolution process of IBDV, our results for segment B showed that the substitution rate for non-vvIBDV lineage was lower than vvIBDV lineage (1.88x10^-4^ per 4.80x10^-3^). These different rates could be explained by several factors. On one hand, segment B from non-vvIBDV lineage has been concomitant with a host for a longer period than vvIBDV lineage. Hence, non-vvIBDV lineage may have a fitness advantage by keeping a narrow population rather than undergoing frequent changes, therefore facilitating a better host-virus equilibrium [75]. On the other hand, the application of different types of vaccines that did not fully protect chickens against infection by vvIBDV strains could have induced a "selective noise" with greater chance for beneficial mutations to accumulate. This “selective noise” allows occasional slips from the first lightest mutational loads (originated by the immune response of the host induced by the vaccination) towards an increase of the weight of mutational loads (given by the *quasispecies* cloud) (Reviewed in [76]) with a consequent increase in the substitution rate.

## Conclusion

In this study, a powerful assessment of a phylogenetic marker included into segment B of the genome of IBDV was performed. This phylogenetic marker showed to be useful for classification and phylodynamic analyses for molecular epidemiology studies regarding segment B of IBDV strains. Evolutionary rates and phylodynamic analyses from B-marker for IBDV showed difference in the mutation rates and the expansion pattern for non-vvIBDV and vvIBDV lineages.

Framed in Cuba, the present work revealed the presence of a novel IBDV natural reassortant between segment A from vvIBDV strains and segment B from non-vvIBDV strains in Cuban IBDV population.

This study also contributed to a better understanding of the emergence of vvIBDV strains, describing molecular epidemiology of IBDV using the state-of-the-art concerning to phylogenetic reconstruction approaches. The present work also proved the presence of a novel natural reassorted strain as possible manifest of change in the genetic structure and stability of the vvIBDV strains. Therefore, it also highlights the need to incorporate the phylogenetically useful information from segment B in the molecular epidemiology studies of IBDV.

## Supporting Information

S1 Fig Structural visualization of the residues translated from B-marker genome region on VP1 crystal. X-ray crystal structures of VP1, crystal structure *2R72* was downloaded from Protein Data Bank; Chimera software v1.6.2 was used for visualization. The residues translated from B-marker genome region were expanded from VP1. Residues belonging to N-terminal domain are denoted in yellow. Residues belonging to F domain are denoted in cyan. The remaining residues translated from B-marker genome region are denoted as heteroatoms. The remains of VP1 structure is maintained in gray.
(TIF)

S2 Fig Molecular analysis of the deduced amino acid sequences for the B-marker of the Cuban field and reference strains. The pattern of the triplet amino acids 145–147 was framed in red rectangle, the position 242 associated with virulence was also framed in red rectangle. Each main lineage (vvIBDV and non-vvIBDV) and Cuban sequences were denoted.
(TIF)

S1 Table IBDV sequences of the hypervariable region of segment A used for classification. (DOCX)

S2 Table Coalescent priors and clock models compared by PS, SS and AICM, the best model was highlighted in boldface. All comparisons were based on equal amounts of independent Monte Carlo samples. SC = strict clock, UCDL = uncorrelated log-normal, UCDE = uncorrelated exponential. Const = constant population size, Exp = exponentially growing population size, Log = Logistic growing population size, BSP = Bayesian skyline plot.
(DOCX)

S3 Table Comparison of topologies obtained for the complete segment B and the B-marker of IBDV using ML and BI methods. **Li**: log-likelihoods, **pKH**: P value for KH normal test (Kishino & Hasegawa 1989), **pRELL**: RELL bootstrap proportions (Kishino & Hasegawa 1989), **pSH**: P value with multiple-comparison correction (MC in Table 1 of Shimodaira & Hasegawa 1999), (-1 for P values means N/A).
(DOCX)
