# Supplementary figures and images for: Evaluation of a Phylogenetic Marker Based on Genomic Segment B of Infectious Bursal Disease Virus: Facilitating a Feasible Incorporation of this Segment to the Molecular Epidemiology Studies for this Viral Agent
Source: PLoS One. 2015 May 6;10(5):e0125853. doi: 10.1371/journal.pone.0125853 (PMC4422720; doi:10.1371/journal.pone.0125853)

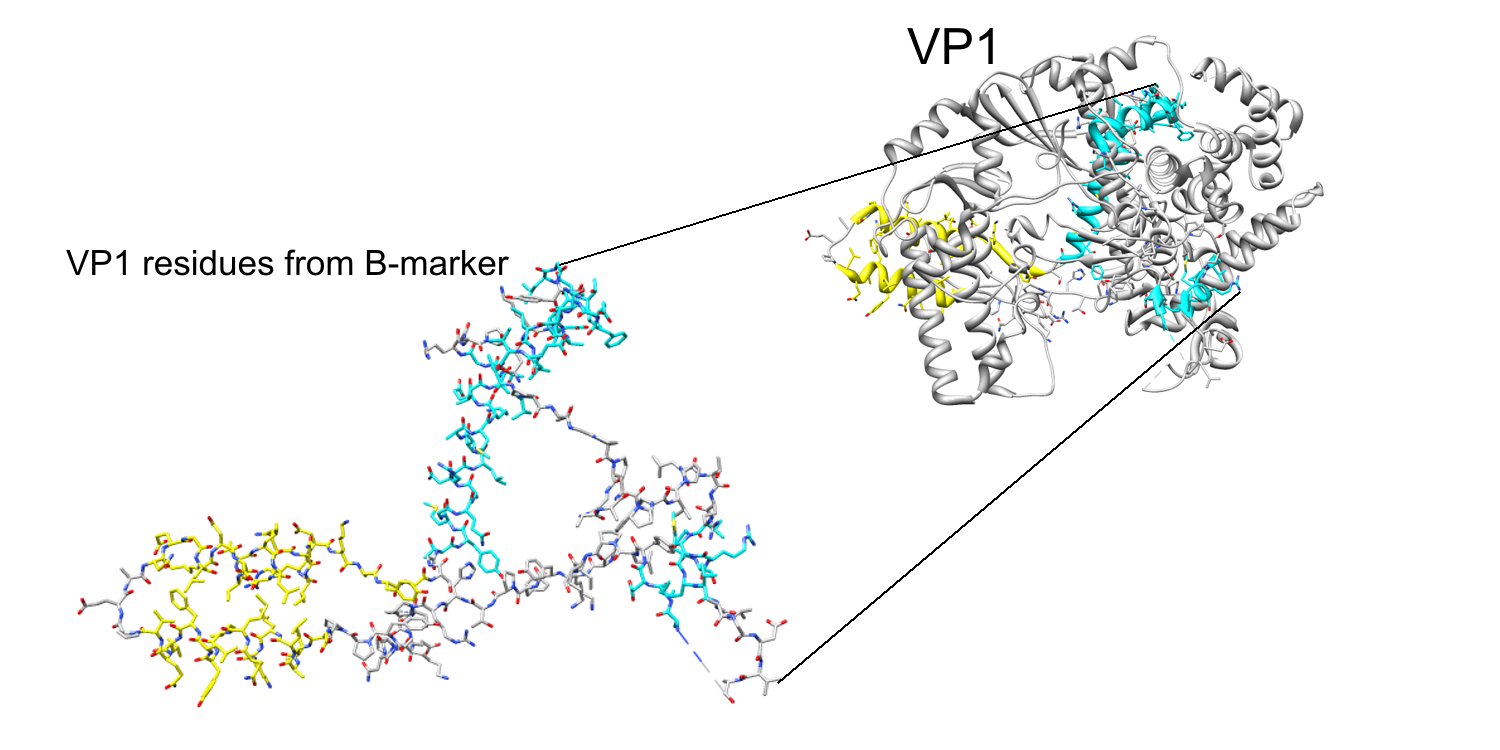

Supplement: S1 Fig — X-ray crystal structures of VP1, crystal structure 2R72 was downloaded from Protein Data Bank; Chimera software v1.6.2 was used for visualization. The residues translated from B-marker genome region were expanded from VP1. Residues belonging to N-terminal domain are denoted in yellow. Residues belonging to F domain are denoted in cyan. The remaining residues translated from B-marker genome region are denoted as heteroatoms. The remains of VP1 structure is maintained in gray. (TIF) [file pone.0125853.s001.tif]

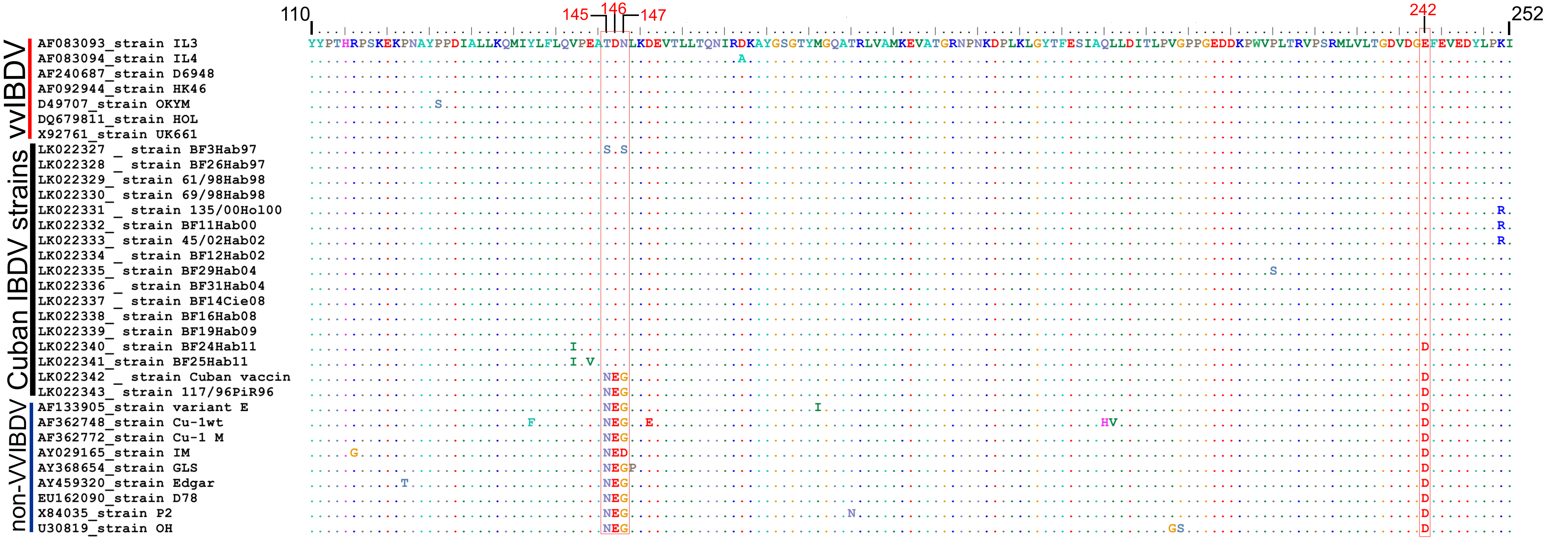

Supplement: S2 Fig — The pattern of the triplet amino acids 145–147 was framed in red rectangle, the position 242 associated with virulence was also framed in red rectangle. Each main lineage (vvIBDV and non-vvIBDV) and Cuban sequences were denoted. (TIF) [file pone.0125853.s002.tif]
