# Supplementary material for: Evaluation of a Phylogenetic Marker Based on Genomic Segment B of Infectious Bursal Disease Virus: Facilitating a Feasible Incorporation of this Segment to the Molecular Epidemiology Studies for this Viral Agent
Source: PLoS One. 2015 May 6;10(5):e0125853. doi: 10.1371/journal.pone.0125853 (PMC4422720; doi:10.1371/journal.pone.0125853)
Supplement: S1 Table — (DOCX) [file pone.0125853.s003.docx]

**Table S1.**

| **Accession number** | **Lineage** | **Strain** | **Year of collection** | **Country** |
| --- | --- | --- | --- | --- |
| AY099456 | vvIBDV | T09 | N/A | Nigeria |
| X84034 | atIBDV | P2 | N/A | Alemania |
| AF240686 | vvIBDV | D6948 | 1987 | Holanda |
| D49706 | vvIBDV | OKYM | 1991 | Japón |
| X92760 | vvIBDV | UK661 | 1989 | Reino Unido |
| AY029166 | cvIBDV | IM | 1967 | EUA |
| M97346 | avIBDV | GLS | 1987 | EUA |
| AY918950 | cvIBDV | Edgar | 1967 | EUA |
| M66722 | serotype 2 | OH | 1982 | EUA |
| EU162087 | atIBDV | D78 | 1978 | Holanda |
| AF322444 | vvIBDV | Tasik | N/A | Indonesia |
| EU184687 | vvIBDV | Cro-Po/00 | N/A | Croacia |
| DQ355819 | cvIBDV | 2512 | N/A | USA |
| AF194428 | atIBDV | CEF94 | N/A | Holanda |
| AJ310185 | atIBDV | CT | N/A | Francia |
| EU184689 | atIBDV | Cro-Pa/98 | N/A | Croacia |
| AB368968 | vvIBDV | KZC-104 | 2004 | Zambia |
| JQ411012 | atIBDV | 903/78 | 1978 | Hungría |
| X54858 | avIBDV | Del-E | 1985 | EUA |
| HF547333 | vvIBDV | 117/96PiR96 | 1996 | Cuba |
| HF547310 | vvIBDV | BF3Hab97 | 1997 | Cuba |
| HF547311 | vvIBDV | BF26Hab97 | 1997 | Cuba |
| HF547314 | vvIBDV | 61/98Hab98 | 1998 | Cuba |
| HF547315 | vvIBDV | 69/98Hab98 | 1998 | Cuba |
| HF547338 | vvIBDV | 135/00Hol00 | 2000 | Cuba |
| HF547317 | vvIBDV | BF11Hab00 | 2000 | Cuba |
| HF547320 | vvIBDV | 45/02Hab02 | 2002 | Cuba |
| HF547321 | vvIBDV | BF12Hab02 | 2002 | Cuba |
| HF547322 | vvIBDV | BF29Hab04 | 2004 | Cuba |
| HF547323 | vvIBDV | BF31Hab04 | 2004 | Cuba |
| HF547337 | vvIBDV | BF14Cie08 | 2008 | Cuba |
| HF547325 | vvIBDV | BF16Hab08 | 2008 | Cuba |
| HF547326 | vvIBDV | BF19Hab09 | 2009 | Cuba |
| HF547347 | vvIBDV | BF24Hab11 | 2011 | Cuba |
| HF547330 | vvIBDV | BF25Hab11 | 2011 | Cuba |
| HF547335 | atIBDV | Gumboro_labiofam | 2011 | Cuba |

| **Accession number** | **Lineage** | **Strain** | **Year of collection** | **Country** |
| --- | --- | --- | --- | --- |
| AY099456 | vvIBDV | T09 | N/A | Nigeria |
| X84034 | atIBDV | P2 | N/A | Alemania |
| AF240686 | vvIBDV | D6948 | 1987 | Holanda |
| D49706 | vvIBDV | OKYM | 1991 | Japón |
| X92760 | vvIBDV | UK661 | 1989 | Reino Unido |
| AY029166 | cvIBDV | IM | 1967 | EUA |
| M97346 | avIBDV | GLS | 1987 | EUA |
| AY918950 | cvIBDV | Edgar | 1967 | EUA |
| M66722 | serotype 2 | OH | 1982 | EUA |
| EU162087 | atIBDV | D78 | 1978 | Holanda |
| AF322444 | vvIBDV | Tasik | NDF | Indonesia |
| EU184687 | vvIBDV | Cro-Po/00 | NDF | Croacia |
| DQ355819 | cvIBDV | 2512 | NDF | USA |
| AF194428 | atIBDV | CEF94 | NDF | Holanda |
| AJ310185 | atIBDV | CT | NDF | Francia |
| EU184689 | atIBDV | Cro-Pa/98 | NDF | Croacia |
| AB368968 | vvIBDV | KZC-104 | 2004 | Zambia |
| JQ411012 | atIBDV | 903/78 | 1978 | Hungría |
| X54858 | avIBDV | Del-E | 1985 | EUA |
| HF547333 | vvIBDV | 117/96PiR96 | 1996 | Cuba |
| HF547310 | vvIBDV | BF3Hab97 | 1997 | Cuba |
| HF547311 | vvIBDV | BF26Hab97 | 1997 | Cuba |
| HF547314 | vvIBDV | 61/98Hab98 | 1998 | Cuba |
| HF547315 | vvIBDV | 69/98Hab98 | 1998 | Cuba |
| HF547338 | vvIBDV | 135/00Hol00 | 2000 | Cuba |
| HF547317 | vvIBDV | BF11Hab00 | 2000 | Cuba |
| HF547320 | vvIBDV | 45/02Hab02 | 2002 | Cuba |
| HF547321 | vvIBDV | BF12Hab02 | 2002 | Cuba |
| HF547322 | vvIBDV | BF29Hab04 | 2004 | Cuba |
| HF547323 | vvIBDV | BF31Hab04 | 2004 | Cuba |
| HF547337 | vvIBDV | BF14Cie08 | 2008 | Cuba |
| HF547325 | vvIBDV | BF16Hab08 | 2008 | Cuba |
| HF547326 | vvIBDV | BF19Hab09 | 2009 | Cuba |
| HF547347 | vvIBDV | BF24Hab11 | 2011 | Cuba |
| HF547330 | vvIBDV | BF25Hab11 | 2011 | Cuba |
| HF547335 | atIBDV | Gumboro_labiofam | 2011 | Cuba |
