# Supplementary material for: Evaluation of a Phylogenetic Marker Based on Genomic Segment B of Infectious Bursal Disease Virus: Facilitating a Feasible Incorporation of this Segment to the Molecular Epidemiology Studies for this Viral Agent
Source: PLoS One. 2015 May 6;10(5):e0125853. doi: 10.1371/journal.pone.0125853 (PMC4422720; doi:10.1371/journal.pone.0125853)
Supplement: S2 Table — All comparisons were based on equal amounts of independent Monte Carlo samples. SC = strict clock, UCDL = uncorrelated log-normal, UCDE = uncorrelated exponential. Const = constant population size, Exp = exponentially growing population size, Log = Logistic growing population size, BSP = Bayesian skyline plot. (DOCX) [file pone.0125853.s004.docx]

# **Table S2.**

| **Clock Model** | **Coalescent** | **ESS** | **AICM** | **Path Sampling (PS)** | **Steping Stone (SS)** |
| --- | --- | --- | --- | --- | --- |
| SC | Const | 8240.25 | 2894.02 | -1515.67 | -1515.79 |
| SC | Exp | 7779.06 | 2893.60 | -1515.27 | -1515.21 |
| SC | Log | 7986.82 | 2892.94 | -1517.89 | -1517.91 |
| SC | BSP | 7099.31 | 2891.69 | -1518.16 | -1517.89 |
| UCDE | Const | 7434.53 | 2887.27 | -1515.69 | -1515.58 |
| UCDE | Exp | 7973.97 | 2890.19 | -1515.30 | -1515.33 |
| UCDE | Log | 8130.80 | 2888.36 | -1518.44 | -1518.41 |
| UCDE | BSP | 7394.11 | 2891.03 | -1517.88 | -1517.76 |
| UCDL | Const | 8148.69 | 2896.08 | -1515.82 | -1515.86 |
| **UCDL** | **Exp** | 7132.99 | 2897.22 | **-1514.87** | **-1514.88** |
| UCDL | Log | 8015.90 | 2896.25 | -1518.17 | -1518.10 |
| UCDL | BSP | 7492.63 | 2896.37 | -1515.91 | -1515.86 |
