# Supplementary material for: Evaluation of a Phylogenetic Marker Based on Genomic Segment B of Infectious Bursal Disease Virus: Facilitating a Feasible Incorporation of this Segment to the Molecular Epidemiology Studies for this Viral Agent
Source: PLoS One. 2015 May 6;10(5):e0125853. doi: 10.1371/journal.pone.0125853 (PMC4422720; doi:10.1371/journal.pone.0125853)
Supplement: S3 Table — Li: log-likelihoods, pKH: P value for KH normal test (Kishino & Hasegawa 1989), pRELL: RELL bootstrap proportions (Kishino & Hasegawa 1989), pSH: P value with multiple-comparison correction (MC in Table 1 of Shimodaira & Hasegawa 1999), (-1 for P values means N/A). (DOCX) [file pone.0125853.s005.docx]

**Table S3.**

| **Tree** | **Li** | **pKH** | **pSH** | **pRELL** |
| --- | --- | --- | --- | --- |
| Complete Segment-B (ML) | -7286.532 | -1 | -1 | 0.689 |
| B-marker (ML) | -7313.042 | 0.08 | 0.458 | 0.017 |
| Complete Segment-B (BI) | -7309.785 | 0.274 | 0.457 | 0.271 |
| B-marker (BI) | -7313.349 | 0.076 | 0.453 | 0.007 |

**Li**: log-likelihoods

**pKH**: P value for KH normal test (Kishino & Hasegawa 1989).

**pRELL**: RELL bootstrap proportions (Kishino & Hasegawa 1989)

**pSH**: P value with multiple-comparison correction (MC in table 1 of Shimodaira & Hasegawa 1999)

(-1 for P values means N/A)
